# Examining the Roles of Emulsion Droplet Size and Surfactant in the Interfacial Instability-Based Fabrication Process of Micellar Nanocrystals

**DOI:** 10.1186/s11671-017-2202-x

**Published:** 2017-06-30

**Authors:** Yuxiang Sun, Ling Mei, Ning Han, Xinyi Ding, Caihao Yu, Wenjuan Yang, Gang Ruan

**Affiliations:** 1 Department of Biomedical Engineering, College of Engineering and Applied Sciences, Nanjing University, Nanjing, China; 2 Collaborative Innovation Center of Chemistry for Life Sciences, Nanjing University, Nanjing, China; 3 Department of Materials Science and Engineering, College of Engineering and Applied Sciences, Nanjing University, Nanjing, China

**Keywords:** Self-assembly, Nanoparticle, Interfacial instability, Microencapsulation, Quantum dot, Magnetic nanoparticle, Biological imaging, Cell separation

## Abstract

The interfacial instability process is an emerging general method to fabricate nanocrystal-encapsulated micelles (also called micellar nanocrystals) for biological detection, imaging, and therapy. The present work utilized fluorescent semiconductor nanocrystals (quantum dots or QDs) as the model nanocrystals to investigate the interfacial instability-based fabrication process of nanocrystal-encapsulated micelles. Our experimental results suggest intricate and intertwined roles of the emulsion droplet size and the surfactant poly (vinyl alcohol) (PVA) used in the fabrication process of QD-encapsulated poly (styrene-b-ethylene glycol) (PS-PEG) micelles. When no PVA is used, no emulsion droplet and thus no micelle is successfully formed; Emulsion droplets with large sizes (~25 μm) result in two types of QD-encapsulated micelles, one of which is colloidally stable QD-encapsulated PS-PEG micelles while the other of which is colloidally unstable QD-encapsulated PVA micelles; In contrast, emulsion droplets with small sizes (~3 μm or smaller) result in only colloidally stable QD-encapsulated PS-PEG micelles. The results obtained in this work not only help to optimize the quality of nanocrystal-encapsulated micelles prepared by the interfacial instability method for biological applications but also offer helpful new knowledge on the interfacial instability process in particular and self-assembly in general.

**Electronic supplementary material:**

The online version of this article (doi:10.1186/s11671-017-2202-x) contains supplementary material, which is available to authorized users.

## Background

The potential of applying nanomaterials, such as fluorescent semiconductor nanocrystals (quantum dots, QDs) [1–3], superparamagnetic iron oxide nanoparticles (SPIONs) [4–6], and gold nanoparticles [7–9], for biomedical detection, imaging and therapy has been well established after nearly two decades of research [10, 11]. Thus, in recent years, the focus of nanobiomaterial research has been shifted from proof-of-concept experiments to mechanistic studies, which aim to obtain insights and systematic understanding on nanomaterial fabrication processes, nanomaterial structure-property relationships as well as nanomaterial-biosystem interactions, and translational research, which aims to identify and solve the key problems in translating nanomaterials to industry and the clinic. The present work focuses on gaining new understanding on an emerging fabrication process, which is known as the interfacial instability method, of micellar nanocrystals, which have become a major class of nanobiomaterials.

A main strategy to solubilize hydrophobic nanomaterials (e.g., QDs, SPIONs, and gold nanoparticles synthesized by the commonly used organic solvent-based high temperature synthesis [12–14]) in water is to use a micelle to encapsulate the hydrophobic nanomaterials [15–17]. A micelle is a classic self-assembly system, in which amphiphilic molecules spontaneously form a core-shell structure (called a micelle) in an aqueous environment, with the hydrophilic segment of the amphiphilic molecules facing outward as the micelle shell and the hydrophobic segment facing inward as the micelle core, to minimize the total energy of the system. Micelles have a long history of applications as cleaning agents and drug delivery systems [18–22], mainly based on the fact that hydrophobic molecules (e.g., oils, many anticancer drugs) can be encapsulated into the hydrophobic cores of the micelles driven primarily by hydrophobic interaction [23]. More recently, micelles have been applied to encapsulate single nanocrystals (with each micelle encapsulating a single nanocrystal) for biomedical imaging and detection [24]. Most recently, quite a few research groups have reported the use of a micelle to encapsulate multiple nanocrystals, for multifunctionality or synergistic effects between the different nanocrystals in a micelle [25–32].

An emerging method to prepare micellar nanocrystals (nanocrystal-encapsulated micelles) is the interfacial instability method [33–35]. The interfacial instability process was first reported in 2008 by Zhu and Hayward to prepare iron oxide nanoparticle-encapsulated micelles [33] and was later used by Ruan and Winter et al. to prepare micelles encapsulating both QDs and SPIONs in 2010 and micelles encapsulating QDs of different fluorescent emission colors in 2011 [25, 26]. The interfacial instability process for preparing QD-encapsulated poly (styrene-b-ethylene glycol) (PS-PEG) micelles involve two main steps: (1) Formation of oil-in-water emulsion droplets. In this emulsion, the oil phase contains hydrophobic QDs and the amphiphilic block copolymer PS-PEG dissolved in a non-polar organic solvent (chloroform in the present work); the aqueous phase contains a surfactant poly (vinyl alcohol) (PVA) dissolved in water; (2) Formation of nanocrystal-encapsulated micelles. Upon evaporation of the organic solvent, the oil/water interface of the emulsion becomes unstable, and hydrophobic interaction drives the system to spontaneously form PS-PEG micelles encapsulating hydrophobic QDs. A simple indicator for successful formation of micelles typically used in the experiments is the dramatic visual transformation of the system from a milky dispersion (emulsion) to a transparent one (micellar nanocrystal dispersion), thanks to the nanometer size (typical diameter 30–40 nm) of the micelles. In Ruan and Winter’s previous experiments with encapsulating QDs into PS-PEG micelles using the interfacial instability process, it was found that, although this process had many positive features, a major problem was the frequently observed great loss of QD fluorescence of the system during the fabrication/storage process, and the cause for the fluorescence loss was unknown. The goals of the present work are twofold: on the one hand, we aim to minimize the fluorescence loss of QD-encapsulated PS-PEG micelles prepared by the interfacial instability process; on the other hand, through the technology optimization process and taking advantage of the fluorescence of QDs as a reporter to follow the fabrication process of QD-containing nanocomposite materials, we aim to gain new understanding on the emerging general process for preparing nanocrystal-encapsulated micelles, i.e., the interfacial instability process. Our results suggest that the emulsion droplet size and the surfactant PVA play key roles in the fabrication process: each emulsion droplet essentially functions as a “micro-reactor” in which interfacial instability and self-assembly “reactions” occur, with the surfactant PVA being required for formation of the “micro-reactors”; Using large “micro-reactor” size (~25 μm) leads to a large portion of colloidally unstable nanocrystal-encapsulated PVA micelles in addition to colloidally stable nanocrystal-encapsulated PS-PEG micelles, while using small “micro-reactor” size (~3 μm or smaller, generated by sonication or electrospray) leads to only colloidally stable nanocrystal-encapsulated PS-PEG micelles.

## Methods

### Materials

Core-shell CdSe/ZnS quantum dots (QDs, emission wavelength 600 nm, covered with octadecylamine) were purchased from Ocean Nanotech. Poly (styrene-b-ethylene glycol) (PS-PEG) and carboxylic acid terminated poly (styrene-b-ethylene glycol) (PS-PEG-COOH) (PS 9.5 k Dalton, PEG 18.0 k Dalton) were purchased from Polymer Source. Poly (vinyl alcohol) (PVA) (molecular weight 13–23 kg/mol, 87–89% hydrolyzed) was purchased from Sigma-Aldrich. Tat peptide (sequence YGRKKRRQRRR) and RGD peptide (Arg-Gly-Asp) were purchased from ChinaPeptides. 1-Ethyl-3-(-3-dimethylaminopropyl) carbodiimide hydrochloride (EDC) and sulfo-NHS were purchased from Sigma-Aldrich. All other chemicals were of reagent grade. The water used for all experiments was double distilled and purified by a Millipore Milli-Q purification system.

### Preparation of Micellar Nanocrystals via the Interfacial Instability Process

In a typical procedure, an oil phase was first formed by mixing QDs (0.1 μM, 0.1 ml) and PS-PEG (10 mg/ml, 20 μl) in the organic solvent chloroform. This was followed by adding an aqueous phase (0.6 ml of water containing 5 mg/ml PVA). An oil-in-water emulsion was formed by either manual shaking (vigorously shaking the mixture by hand for 1 min) or sonication (sonicating the mixture in a ShuMei KQ218 bath sonicator for 30 s). In some experiments, electrospray was used to generate ultrafine emulsion droplets for the interfacial instability process [35]. The different treatments were used to generate emulsion droplets with different sizes for studying the effects of droplet size: ~25 μm (in diameter) droplets were formed by manual shaking, ~3 μm (in diameter) droplets were formed by sonication, and a few hundred nanometers to a few micrometer (in diameter) droplets were formed by electrospray. The emulsion was diluted by an additional factor of four with ultrapure water (2.4 ml). The emulsion was left in a chemical fume hood with magnetic stirring at 100 rpm to allow evaporation of chloroform, leading to formation of micellar QDs. A visible transition in appearance from a milky dispersion to a transparent one was indicative of successful micelle formation.

When tetrahydrofuran (THF) was used as the organic solvent, an oil phase was first formed by mixing QDs (0.1 μM, 1 ml) and PS-PEG (10 mg/ml, 0.2 ml) in THF. Deionized water was added to the solution in a drop-wise manner (1 drop/20 s) until the water content reached 50% *v*/*v*. The solution was then mixed by votexing for 10–15 min and was then dialyzed against deionized water for 2 days to remove THF (molecular weight cutoff 100,000 Dalton).

When electrospray was used to generate droplets for the interfacial instability process, the operation was as follows [35]. A coaxial electrospray configuration was used. The inner capillary needle was a 27 gauge (outer diameter 500 μm; inner diameter 300 μm) stainless steel capillary, and the outer needle was a 20 gauge (outer diameter 1000 μm; inner diameter 500 μm) stainless steel three-way connector. The nozzle tip was positioned 0.8 cm above a grounded steel ring and 10 cm above a glass collection dish. An oil phase was formed by mixing QDs and PS-PEG and was then delivered to the inner stainless steel capillary at a flow rate of 0.6 ml/h using a syringe pump (SPLab01, Shenzhen, China). The concentrations of PS-PEG and QDs in the oil phase were 5 mg/ml and 0.2 μM, respectively. An aqueous phase was prepared by dissolving PVA in deionized H_2_O at 40 mg/ml. The aqueous solution was delivered to the outer annulus of the coaxial needle at a flow rate of 1.5 ml/h using a second syringe pump (SPLab01, Shenzhen, China). Typically at a voltage in the range of 6–7 kV, a concave cone-jet (Taylor cone) was observed at the tip of the coaxial nozzle. A glass collection dish containing 10 ml deionized water was placed below the nozzle tip to collect droplets. The electrospray time (after a stable Taylor cone was formed) was typically 30–90 min. This was followed by further evaporation in the chemical fume hood overnight. Finally, the dispersion in the glass collection dish was transferred to a 15 ml centrifuge tube for characterizations.

### Characterizations of the Physical Properties of Micellar QDs

The morphology of micellar QDs was characterized by transmission electron microscopy (TEM, JEOL JEM-2100 (HR)), and all samples investigated by TEM in the present work were negatively stained by 1% of phosphotungstic acid (PTA). Particle size was characterized by TEM or dynamic light scattering (DLS). Fluorescent spectra were obtained by a Hitachi F-4600 Fluorescent spectrophotometer.

### Cytotoxicity of Nanomaterials

Cytotoxicity study was performed on three well-characterized human cancer cell lines, namely, A549 (alveolar basal epithelial), MCF-7 (breast), and HeLa (cervix) cells (purchased from KeyGen Biotech, China). The cells were maintained with cultured DMEM with 10% fetal bovine serum and antibiotics (penicillin/streptomycin) in a humid incubator (37 °C and 5% CO_2_). For cytotoxicity evaluation, cells were seeded onto 96-well plates in 200 μl of medium for 24 h. Then, the cells were incubated with different concentrations of micellar QDs in fresh culture medium at 37 °C in 5% CO_2_ atmosphere. After 24 h incubation, the culture media with dispersed micellar QDs were removed and the MTT assay was applied according to the manufacturer’s protocol. Finally, the optical absorbance in each well was measured at 570 nm in a microtiter plate reader.

### Conjugation of QD-Encapsulated PS-PEG-COOH Micelles with Peptides

PS-PEG-COOH micelles were prepared with the above-described interfacial instability procedure, with PS-PEG-COOH molecules being used instead of PS-PEG ones. Conjugation with Tat peptide or RGD peptide was then conducted via the EDC/sulfo-NHS method. To activate the carboxyl groups of micelles, 0.3 ml of 0.1 M MES buffer solution containing 2 mg/ml EDC and 5 mg/ml sulfo-NHS was added to the micelle dispersion (3 ml) and reacted without stirring for 30 min at room temperature. The extra EDC and sulfo-NHS were then removed by using a 30-kD ultrafiltration tube (centrifugation at 10 krpm for 5 min), and the obtained dispersion was re-suspended in PBS (1 ml). Subsequently, 50 μl of Tat peptide (2 mg/ml in PBS) or 50 μl of RGD peptide (0.5 mg/ml in PBS) was added and reacted for 12 h at 4 °C, respectively. The obtained peptide-conjugated PS-PEG micellar QD dispersion was purified by using a 50-kD ultrafiltration tube (centrifugation at 10 krpm for 5 min) for three times to remove the extra peptide molecules and re-suspended in PBS (1 ml).

### Live Cell Imaging

Live cell imaging was used to study the cellular internalization and intracellular transport of Tat peptide-conjugated PS-PEG micellar QDs. HeLa cells (purchased from KeyGen Biotech, China) were seeded on glass-bottom tissue culture plates at an initial confluency of 20% (seeding density 1 × 10^5^ cells/ml) in 600 μl of medium (DMEM + 10% fetal bovine serum) and were cultured for 40 h in 5% CO_2_ at 37 °C. Tat peptide-conjugated PS-PEG micellar QDs (10 nM of QDs in cell culture medium) were then added. After being incubated with the micellar QDs for 1 h, the cells were washed twice with fresh culture medium to remove free micellar QDs (the washing step was done in order that the starting time of the intracellular transport of the internalized micellar QDs could be roughly the same for all the nanoparticles added). After 6 h, each plate of cells was imaged by a live cell imaging system, which consists of a cell incubation chamber (IX3W, Tokai Hit), an epi-fluorescent microscope (IX-83, Olympus, with halogen lamp as the light source), a spinning disk confocal system (Andor) and an electron multiplying charge-coupled device (EMCCD) camera (Evolve 512, Photometrics). The live cell confocal imaging system used here permits spinning-disk confocal imaging of live cells cultured on the microscope stage, which is particularly useful for studying the cellular transport process. By keeping the live cells cultured on the microscope stage, one could ensure that the natural biological process is monitored with minimal disturbance. To counter-stain the cell nucleus, right before imaging (at a particular time point of cellular transport), the fluorescent dye Hoechst 33342 (5 μM in cell culture medium) was incubated with live cells for 20 min.

Live cell imaging was also applied to study the specific binding of RGD peptide-conjugated PS-PEG micellar QDs with α_*v*_β_3_-integrin molecules, using an α_*v*_β_3_-integrin over-expressed cell line (U87 MG cells, purchased from KeyGen Biotech, China) versus a cell line without α_*v*_β_3_-integrin over-expression (MCF-7 cells, purchased from KeyGen Biotech, China). The above cellular imaging protocol used for Tat peptide-conjugated PS-PEG micellar QDs was adopted, with the main modification being that the concentration used for RGD peptide-conjugated micellar QDs was 100 nM (QDs in cell culture medium).

## Results and Discussion

We and others recently introduced the interfacial instability method to encapsulate nanocrystals to form composite nanoparticles for biological applications. However, we frequently encountered irreproducible and sometimes conflicting results on fluorescence intensity of QDs (QDs were used as the model of nanocrystals here). This issue needs to be addressed for translation to industry and the clinic. Many factors (e.g., solvent, polymer, temperature, “micro-reactor” size) involved throughout the fabrication process could lead to fluorescence loss and irreproducible results. We have investigated the various factors involved and have found that the “micro-reactor” (emulsion droplet) size is a key factor in this regard, with the use of surfactant PVA being a closely related factor. Below, we primarily describe the results on the effects of emulsion droplet size as well as the surfactant PVA.

**Fig. 1:**
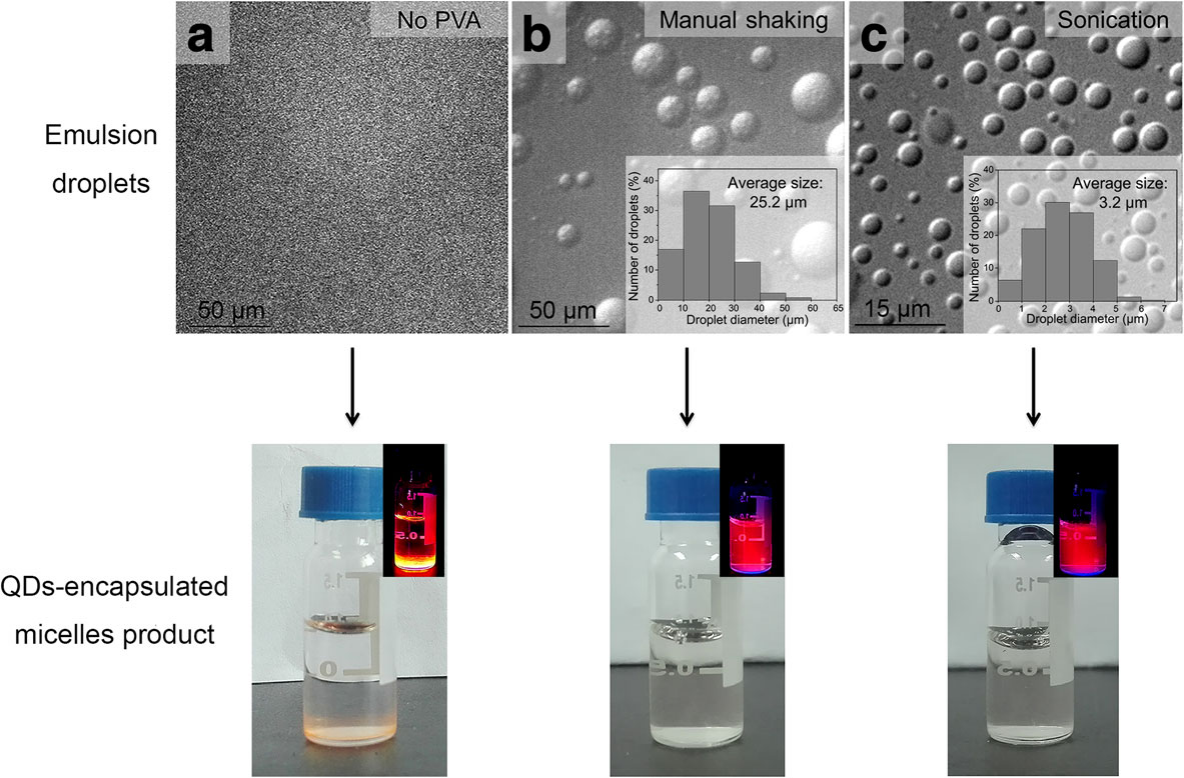
Visual observation of the emulsion droplets and the resulted QD-encapsulated micelles after organic solvent evaporation. **a** No PVA was used. Few to no emulsion droplets were formed (*top image*); few to no QD-encapsulated micelles were formed upon organic solvent removal (*bottom image*, the *inset* shows corresponding fluorescent image using a hand-held UV lamp to excite the *red* QD fluorescence). **b** Manual shaking was used to form emulsion droplets. ~25 μm emulsion droplets were formed (*top image*, the *inset* shows the droplet size measurement result from image analysis of 500 droplets). Additionally, the size variation due to different shaking times was found to be minimal (Fig. S1). Upon organic solvent removal a transparent and homogeneous dispersion was formed, indicating successful formation of nanocrystal-encapsulated micelles (*bottom image*, the *inset* shows corresponding fluorescent image using a hand-held UV lamp to excite the *red* QD fluorescence). **c** Bath sonication was used to form emulsion droplets. ~3 μm emulsion droplets were formed (*top image*, the *inset* shows the droplet size measurement result from image analysis of 500 droplets). Additionally, the size variation due to different shaking times found to be minimal (Fig. S1). Upon organic solvent removal, a transparent and homogenous dispersion was formed, indicating successful formation of nanocrystal-encapsulated micelles (*bottom image*, the *inset* shows corresponding fluorescent image using a hand-held UV lamp to excite the *red* QD fluorescence). To analyze the size of emulsion droplets of a particular sample, firstly, a light microscopy image of the emulsion droplets was taken, and subsequently, the diameters of ~500 droplets were measured by the free software ImageJ to obtain the average size and size distribution of the emulsion droplets of the sample

We compared the effects of two different methods of emulsification with greatly different mechanical strengths, namely, manual shaking (vigorously shaking the mixture manually) and bath sonication (sonicating the mixture in a bath sonicator). We found that both of these two methods could eventually lead to transparent and homogeneous dispersions after evaporation of organic solvent, indicating successful nanocrystal-encapsulated micelle formation (Fig. 1b, c, bottom). Because transparent and homogeneous visual appearance is commonly used as a simple and convenient indicator of successful micelle formation in the interfacial instability-based fabrication process, the potential impact of different emulsification methods on the micelle product was previously overlooked. We used light microscopy to examine the sizes of emulsion droplets generated by these two different emulsification methods, respectively, and found that the manual shaking method led to ~25 μm (in diameter) droplets, while the bath sonication method led to ~3 μm (in diameter) ones (Fig. 1b, c, top). Approximately the sizes of 500 droplets were measured for each sample using light microscopy images to obtain the average size and size distribution. Statistical analysis (Student’s *t* test) shows that the difference between the average size of droplets formed by manual shaking (~25 μm) and that by sonication (~3 μm) was statistically significant (*P* < 0.001). Importantly, we also performed control experiments to confirm that these two different emulsification methods consistently produce emulsion droplet sizes in the abovementioned two different size ranges, respectively: manual shaking with several different time durations (0.5, 1, 2, and 3 min) all resulted in ~25 μm emulsion droplets (with similar size distribution) and bath sonication with several different time durations (0.5, 1, and 2 min) all resulted in ~3 μm emulsion droplets (with similar size distribution, Additional file 1: Figure S1).

Furthermore, we also conducted emulsification treatment in the absence of the surfactant PVA and found that virtually no emulsion droplets were successfully formed, judging from the light microscopy result (Fig. 1a, top), and virtually, no micelles were successfully formed, judging from the observation of nearly complete phase separation (QD precipitation) in the final product, i.e., failure to form micelle product (Fig. 1a, bottom). The results of Fig. 1a suggested that the surfactant PVA is required in the interfacial instability process for successful formation of emulsion droplets (as the “micro-reactors”) and of micelles (as the final products). This is non-trivial because it suggests that, although PS-PEG is also amphiphilic in nature, the presence of PS-PEG alone (without the presence of PVA) in the system cannot give the emulsion droplets needed for the interfacial instability process.

**Fig. 2:**
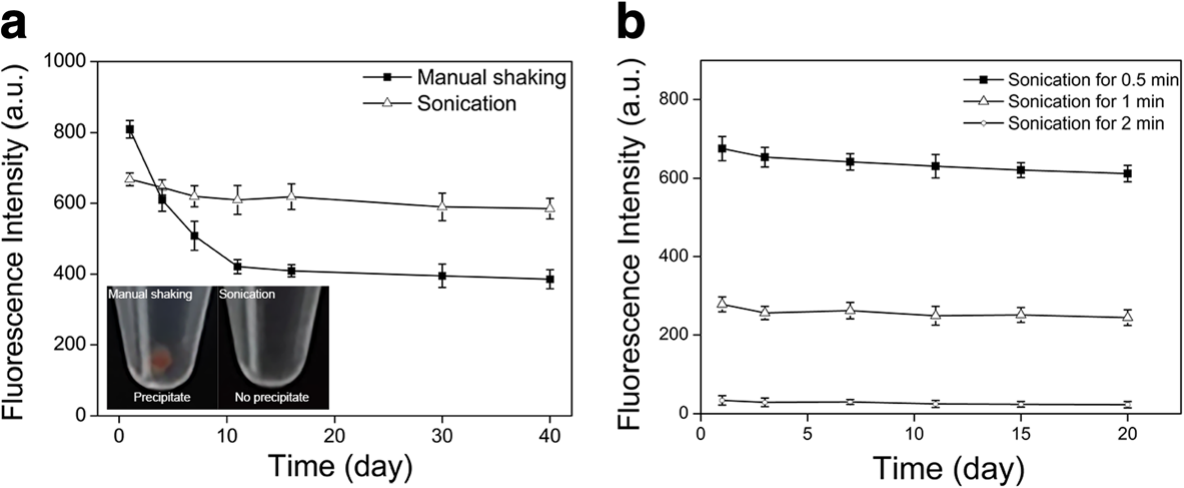
Fluorescence stability of QD-encapsulated micelles fabricated by the interfacial instability process. **a** Change of fluorescence intensity (measured by fluorescent spectroscopy) over time for QD-encapsulated micelles, with the emulsion droplets formed by either manual shaking (for 1 min, i.e., ~25 μm droplets) or sonication (for 30 s, i.e., ~3 μm droplets), respectively. The *insets* are images of the QD-encapsulated micelle dispersions after 10 days of storage at 4 °C with the emulsion droplets formed by either manual shaking (for 1 min, i.e., ~25 μm droplets) or sonication (for 30 s, i.e., ~3 μm droplets), respectively. Panel **a** indicates that one cause of loss of fluorescence of QD-encapsulated micelles is the presence of colloidally unstable QD-encapsulated micelles. **b** Change of fluorescence intensity (measured by fluorescent spectroscopy) over time for QD-encapsulated micelles with the emulsion droplets formed by sonication (i.e., ~3 μm droplets) for three different sonication treatment time durations. Panel **b** indicates that one cause of loss of fluorescence of QD-encapsulated micelles is QD surface defects generated by strong and prolonged mechanical treatment

Although the product dispersions appeared to be transparent and homogeneous right after they were formed (TEM and DLS characterization results showed spherical and monodispersed QD-encapsulated micelles, Additional file 2: Figure S2), the difference in emulsion droplet sizes given by the above two different emulsification methods, namely, manual shaking and sonication, was found to lead to great difference in the micellar nanocrystals products. We measured and followed the fluorescent intensity change of the micellar QDs (QD-encapsulated micelles) formed by manual shaking and sonication, respectively, over a time period of 40 days at 4 °C. We found that, for the micellar, QDs formed by manual shaking (for 1 min, formed from ~25 μm emulsion droplets), although the fluorescent intensity (measured by fluorescent spectroscopy) was maintained during the micelle formation process, over time (~10 days) the fluorescence intensity of the micellar QDs decreased gradually to only approximately 50% of the original fluorescent intensity level, and remained steady afterwards (Fig. 2a). In contrast, the micellar QDs formed by sonication (for 30 s, formed from ~3 μm emulsion droplets) largely maintained fluorescent intensity (measured by fluorescent spectroscopy) over the entire time period (Fig. 2a). Further, we used naked eyes to observe the bottom part of the micellar QD dispersions formed by manual shaking and sonication, respectively, after the samples were left standing for 10 days at 4 °C. In the bottom part of the micellar, QD dispersions formed by manual shaking (for 1 min) after 10 days of storage, visible precipitate was observed by naked eyes (Fig. 2a, inset). In contrast, in the bottom part of the micellar QD dispersions formed by sonication (for 30 s) after 10 days of storage, no visible precipitate was observed by naked eyes (Fig. 2a, inset). These results suggest that, although both emulsification methods could lead to successful formation of QD-encapsulated micelles with similar encapsulation efficiency, a large portion of the QD-encapsulated micelles formed by larger emulsion droplets (~25 μm, produced by manual shaking for 1 min) was colloidally unstable and over time resulted in precipitation and thus fluorescence loss from the dispersion, while all of the QD-encapsulated micelles formed by smaller emulsion droplets (~3 μm, produced by bath sonication for 30 s) were colloidally stable and thus maintained fluorescence for long period of time (TEM study of the dispersion after 10 day storage also showed well-maintained morphology of micellar nanocrystals, as shown in Additional file 3: Figure S3). In addition, we found that, when the sonication time was increased from 30 s to 1 and 2 min to form emulsion droplets, the fluorescent intensity was drastically reduced, although the QD-encapsulated micelles were colloidally stable for long period of time for the different sonication treatment time durations judging by the stable fluorescent intensity throughout the storage time (Fig. 2b). The fluorescence loss shown in Fig. 2b was likely caused by generation of surface defects on QDs by the strong and prolonged mechanical treatment. In a control experiment, the fluorescent intensity of hydrophobic QDs dissolved in chloroform was also found to decrease gradually under sonication treatment with increased sonication time (Additional file 4: Figure S4), which supports this proposed cause of fluorescence loss. Together, Fig. 2a, b reveals the two main mechanisms of fluorescence loss in the QD-encapsulated micelle system fabricated by the interfacial instability process, namely, colloidally unstable QD-encapsulated micelles and mechanical treatment-generated QD surface defects.

**Fig. 3:**
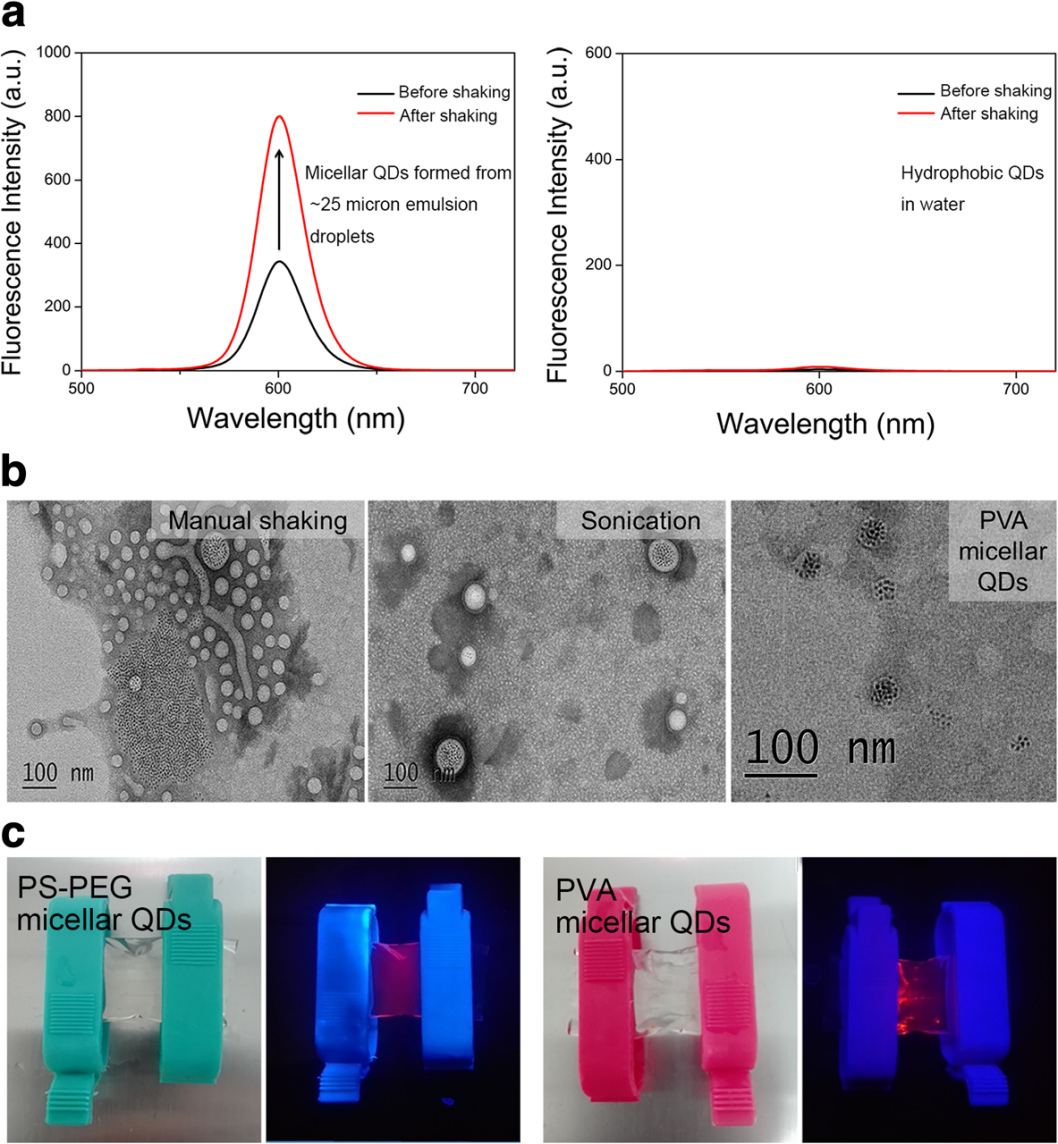
Examining the colloidally unstable part of the micellar QDs. **a** *Left*, the disappearance of fluorescence of the colloidally unstable part of the micellar QDs from the dispersion after 10-day storage could be resumed after the dispersion was shaken. *Right*, the fluorescence of hydrophobic QDs was not detected in water because they could not be dispersed in water. **b** *Left* and *middle* are the TEM images of the bottom portions of the micellar QD dispersions formed from manual shaking for 1 min (i.e., ~25 μm emulsion droplets) and sonication for 30 s (~3 μm emulsion droplets), respectively, after 10-day storage. *Right*, TEM image of PVA micellar QDs. **c** Dialysis (against water) treatment on PS-PEG micellar QDs (*left*) and PVA micellar QDs (*right*), respectively. A hand-held UV lamp was used to excite the *red* QD fluorescence

Among these two mechanisms of fluorescence loss, although it is well known that QD fluorescence loss can be caused by damage to QD surface, the result that part of the micelles being colloidally unstable was a surprise to us. Thus, we conducted further study on this particular mechanism. We first asked the question whether or not the abovementioned precipitated QDs were indeed encapsulated in micelles (or micelle-like assembly structures). We found that simply shaking the dispersion with these precipitates could lead to return of the fluorescent intensity of the dispersion to the original level (Fig. 3a, left); in contrast, a control study on using the same treatment for hydrophobic QDs precipitated in water showed no such increase of fluorescent intensity (Fig. 3a, right). In addition, transmission electron microscopy (TEM) images of the bottom part of the product samples formed by manual shaking after 10 days of storage showed a great number of QDs clustered in large spherical and non-spherical structures (Fig. 3b, left); in contrast, in the bottom part of the product samples formed by sonication after 10 days of storage, the corresponding TEM images only showed a small number of QDs clustered in spherical structure (Fig. 3b, middle). These results thus show that the precipitated QDs were indeed encapsulated in micelles or micelle-like assembly structures, i.e., these QDs were not naked hydrophobic QDs. We then asked the question what is the chemical nature of these colloidally unstable micelles (or micelle-like assembly structures). Because a PS-PEG micelle should be colloidally stable, we hypothesized that the unstable micelles (or micelle-like assembly structures) were formed by the surfactant PVA. This hypothesis is supported by the following two lines of experimental evidence. First, we found that using PVA without PS-PEG in the interfacial instability process could indeed result in micelles encapsulating QDs (Fig. 3b, right). Second, we performed dialysis experiments on PS-PEG micellar QDs and PVA micellar QDs, respectively, for comparison. After dialysis treatment (with the molecular weight cutoff of the dialysis bag being 200 kD, which is larger than the molecular weights of PVA and PS-PEG) against pure water, the PS-PEG micellar QDs remained colloidally stable, judging by the fact that the QD fluorescence remained homogenous in the dispersion (Fig. 3c, left). In stark contrast, the PVA micellar QD dispersion led to clearly visible fluorescent aggregates after nearly identical dialysis treatment as above (Fig. 3c, right). As the dialysis experiment could be considered as mimicking the dilution treatment that micelle-based nanomaterials would encounter once introduced to an in vivo environment, our dialysis experimental results indicate that the QD-encapsulated PVA micelles would become colloidally unstable in vivo. Therefore, the results shown in Fig. 3c suggest that the fluorescence loss from using large emulsion droplets (~25 μm, produced by manual shaking) is caused by colloidally unstable PVA micelles (or other micelle-like assembly structures) encapsulating QDs.

**Fig. 4:**
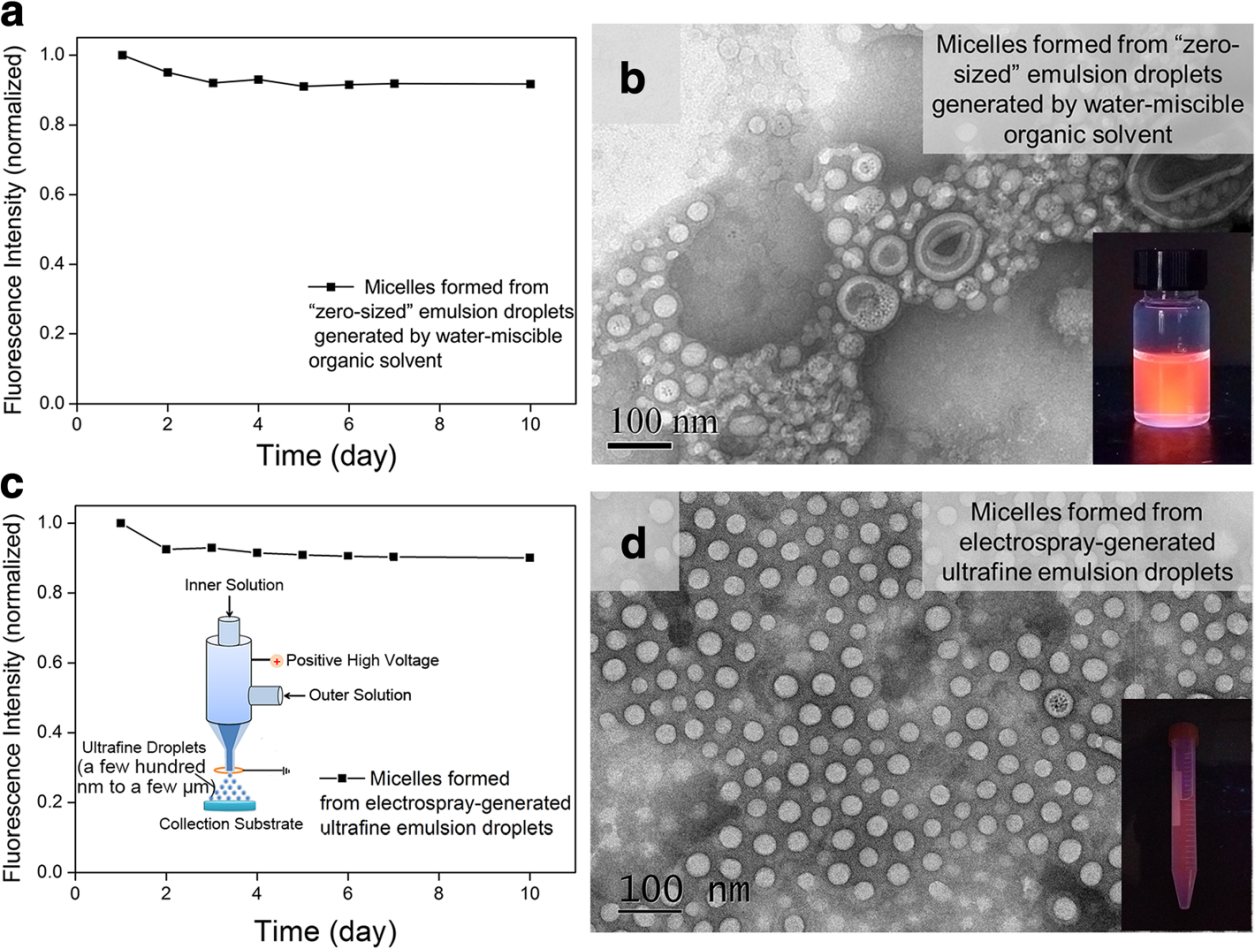
Using additional methods to form small emulsion droplets to confirm the importance of droplet size. **a**, **b** Using water-miscible THF as the oil phase solvent (without using PVA) led to micellar QDs with stable fluorescence (**a**) and irregular micelle shapes (**b**). **c**, **d** Using electrospray (with PVA) to form droplets led to micellar QDs with stable fluorescence (**c**) and regular micelle shape (**d**)

Further, we conducted two additional experiments to confirm the roles of emulsion droplet size and the surfactant PVA. In the first experiment, we used a water-miscible organic solvent tetrahydrofuran (THF) instead of the water immiscible organic solvent chloroform. In this case, the “emulsion droplet” size could be considered as zero, and the surfactant PVA was not used because it was not needed to facilitate the mixing of oil phase with water phase. It was found that the fabrication process produced QD-encapsulated micelles with stable fluorescence (Fig. 4a), which is consistent with the result that small emulsion droplets lead to colloidally stable QD-encapsulated micelles and stable fluorescence. In addition, it was observed that the micelles formed by this process (with THF, without PVA) had large size distribution and some of the formed micelles even had non-spherical shapes (Fig. 4b). This indicates that “zero droplet size” could lead to poorly controlled micelle size and shape (although the formed micelles are colloidally stable). Thus, the results of the “zero emulsion droplet size” experiment (with the water-miscible THF as the organic solvent), on the one hand, are consistent with the finding that smaller emulsion droplets lead to colloidally stable micellar nanocrystals (judging by the stable fluorescence given by “zero-sized emulsion droplets”), and on the other hand, indicate the advantage of having an emulsion droplet (with non-zero droplet size) compared with no emulsion droplet at all (“zero-droplet size”, which gives poor micelle morphology). In the second experiment, we used electrospray, which is known to give ultrafine and uniform droplets with the typical droplet size range being a few hundred nanometers to a few micrometers (smaller than what the sonication treatment generates), as the method to produce emulsion droplets (PVA was used in this case) [35–39]. It was found that this method led to micellar QDs with stable fluorescence and well-controlled micelle size and shape (Fig. 4c, d). It should be mentioned that electrospray typically can only produce droplet sizes smaller than what the sonication treatment gives (i.e., a few hundred nanometers to a few micrometers). Thus, to study the effect of larger emulsion droplet size, in this work, we used another mechanical treatment method, i.e., manual shaking, to give larger droplets (~25 μm). The actual size of electrospray-generated droplets is difficult to be obtained by direct imaging (for example, the size of electrospray-generated oil-in-water emulsion droplets would change greatly upon entering the large volume of water phase in the collection container due to aggregation and fusion, and the typical sub-micrometer size of electrospray-generated droplets is approaching the diffraction limit of optical microscopy), but could be theoretically calculated or experimentally measured by methods that characterize aerodynamic mobility as done previously in the literature.

**Fig. 5:**
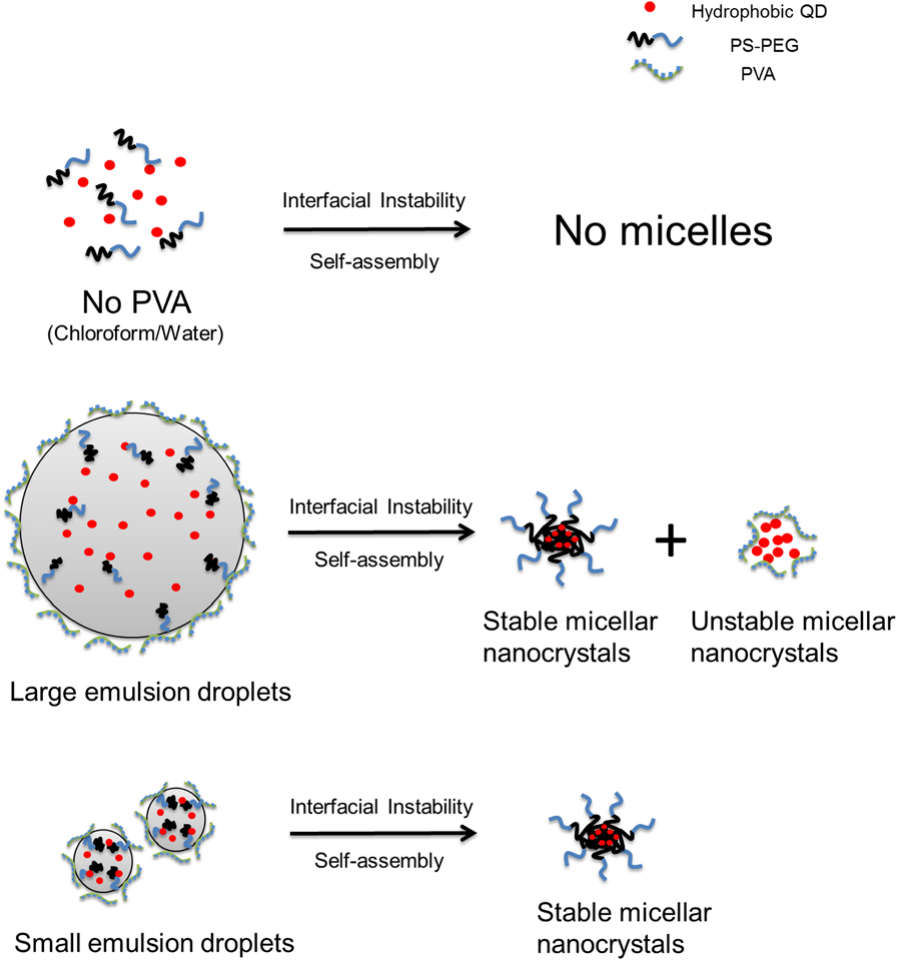
Roles of emulsion droplet size and surfactant in the interfacial instability-based fabrication of micellar nanocrystals

Figure 5 presents a schematic to summarize our results and insights on the roles of emulsion droplets and the surfactant PVA in the interfacial instability-based fabrication process of nanocrystals-encapsulated micelles (with QDs as the model for nanocrystals). Each oil-in-water emulsion droplet serves as a “micro-reactor” for the interfacial instability-mediated self-assembly “reaction.” When no PVA (surfactant) is used, emulsion droplet does not form, and thus, no micelle is formed. When the emulsion droplet is large in size (~25 μm), only a part of the QDs (approximately 50%, based on the remaining fluorescence intensity in the dispersion after 10-day storage, Fig. 2a) in the droplet get encapsulated in PS-PEG (an amphiphilic block copolymer) micelles, which are colloidally stable, while the other part of the QDs get encapsulated in PVA (also an amphiphilic polymer, but not a block copolymer) micelles, which are colloidally unstable. When the emulsion droplet is small in size (~3 μm or smaller), nearly all QDs (based on the remaining fluorescence intensity in the dispersion after 10-day storage, combined with comparison of fluorescent intensity with hydrophobic QDs undergoing similar mechanical treatment, Fig. 2a, Fig. 4a, c, and Additional file 4: Fig. S4) in the droplet get encapsulated in stable PS-PEG micelles. Thus, the roles of emulsion droplets and the surfactant PVA in the interfacial instability-based fabrication process of nanocrystal-encapsulated micelles are intricate and intertwined, particularly in the context of biological applications: the surfactant PVA is required for successful formation of emulsion droplets and micelle products, and yet it is also responsible for formation of the colloidally unstable part of nanocrystal-encapsulated micelles, which would be detrimental in a number of biological applications; and the key to avoid the colloidally unstable nanocrystal-encapsulated PVA micelles is to use emulsion droplets small in size (~3 μm or smaller).

In addition, it should be mentioned that, for a well-dispersed oil-in-water emulsion to form, a surfactant is often required to lower the surface tension between the oil phase and water phase, and PVA was selected here as the surfactant because it was applied in nearly all the previous works on using the interfacial instability method to fabricate micelles [25, 26, 33–35]. We cannot rule out the possibility that other surfactants could give different results. Examining the effects of different types of surfactant would be part of the future studies.

**Fig. 6:**
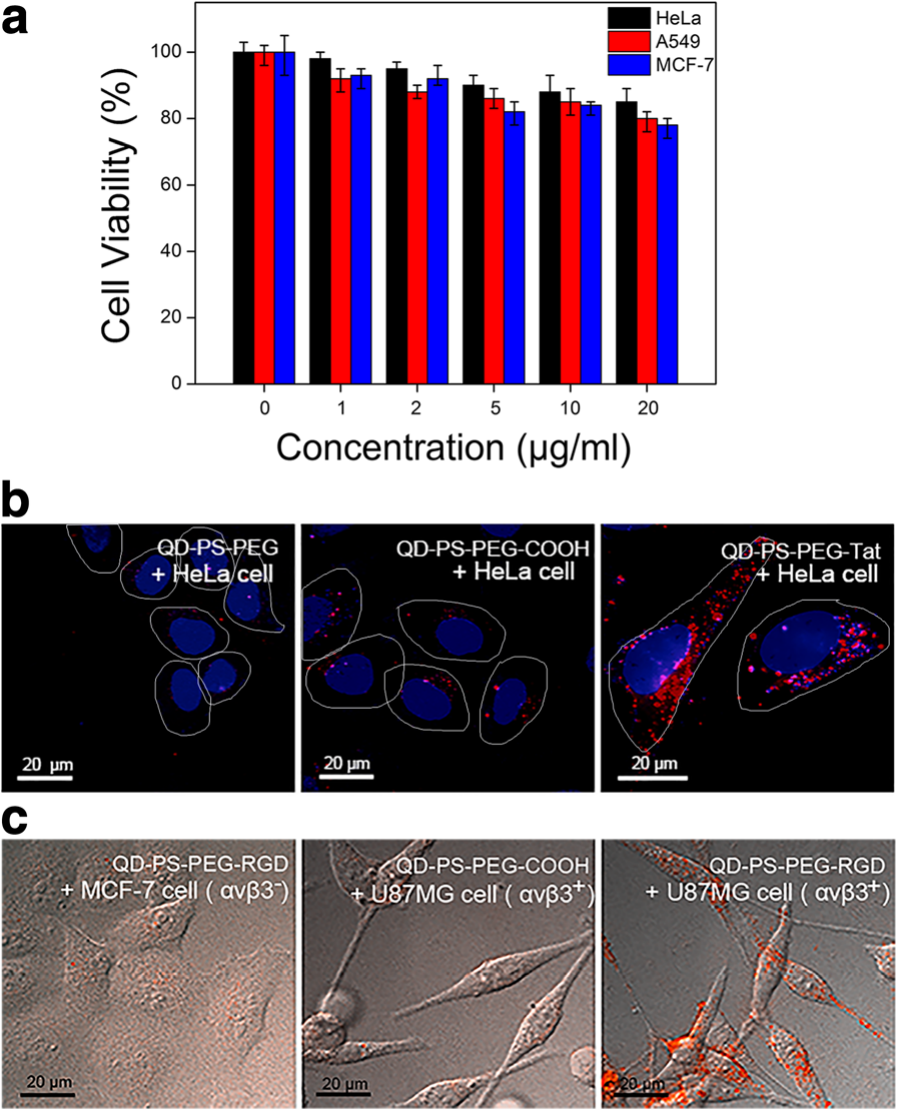
Interactions of PS-PEG micellar QDs (prepared by using sonication 30 s in the interfacial instability method) with biological cells. **a** PS-PEG micellar QDs were fairly biocompatible judging from the MTT cytotoxicity assay results. The concentrations were based on the amounts of QDs used. **b** Tat peptide-conjugated PS-PEG micellar QDs can be internalized by live cells. **c** RGD peptide-conjugated PS-PEG micellar QDs can specifically recognize and bind with the α_*v*_β_3_-integrin molecules over-expressed on U87MG cells (*right image*). In comparison, in the absence of α_*v*_β_3_-integrin over-expression (MCF-7 cells, *left image*) or Tat peptide (PS-PEG-COOH micellar QDs, *middle image*), no significant binding (QD fluorescence) was observed. The *red* fluorescence was from QDs. The cell nucleus was stained by the *blue* fluorescent dye Hoechst 33342. Cell periphery is shown by *white line* (from the corresponding bright field microscopy images)

Finally, we performed proof-of-concept biological experiments using live cells to demonstrate that our micellar nanocrystal products (with the emulsion droplets formed from sonication treatment for 30 s) are (1) fairly biocompatible, (2) can be functionalized with biological molecules, (3) can be introduced into live cells, and (4) if conjugated with biological targeting molecules, can bind with specific biological targets (Fig. 6). Cytotoxicity studies by MTT assay showed that the QD-encapsulated PS-PEG micelles had fairly low cytotoxicity in three different cell lines compared with the negative control (cultured cells in the absence of nanomaterials added, i.e., concentration being zero) (Fig. 6a). To bio-functionalize QD-encapsulated PS-PEG micelles, in the micelle fabrication process the PS-PEG molecules were replaced with PS-PEG-COOH molecules, the latter of which could then be conjugated with a wide spectrum of biomolecules (e.g., peptides, nucleic acids, and antibodies) via well-established bioconjugation methods. To show that QD-encapsulated PS-PEG micelles can be introduced into live cells, the micelles were conjugated with Tat peptide, which is derived from HIV virus and is known to be able to introduce a variety of nanomaterials into live cells with high efficiency and low toxicity [40–42]. The thus-formed Tat peptide-conjugated PS-PEG micellar QDs were then incubated with HeLa cells, and live cell confocal imaging was conducted to study the cellular transport of the fluorescent nanomaterials. The live cell confocal imaging system used here permits spinning-disk confocal imaging of live cells cultured on the microscope stage, ensuring that the natural transport process is followed with minimal disturbance. HeLa cells were selected here because this cell line was used in the first tracking study of the cellular transport of Tat peptide-conjugated QDs by Ruan et al. [42]. It was found that, 6 h after the first contact of Tat peptide-conjugated PS-PEG micellar QDs with the cells, many of the Tat peptide-conjugated PS-PEG micellar QDs had been internalized by the cells, judging by the composite confocal images to show the positions of QDs, cell nucleus and cell periphery, and the cellular uptake level was much higher than that without the assistance of Tat peptide (Fig. 6b). Imaging of the change of QD distribution at different time points of cellular transport for the Tat peptide-conjugated PS-PEG micellar QDs indicated that, after entering the cells, they were gradually accumulated at a perinuclear region (Additional file 5: Fig. S5). Additional file 6: video 1 shows a three-dimensional reconstructured image of the distribution of Tat peptide-conjugated PS-PEG micellar QDs in the cell at the time point of 24 h, which further confirms cellular internalization and perinuclear accumulation. The behavior of the cellular transport of Tat peptide-conjugated PS-PEG micellar QDs is consistent with that of Tat peptide-conjugated QDs previously reported in the literature [42]. Further, to show that QD-encapsulated PS-PEG micelles can be modified (bio-functionalized) to bind with specific biological targets via ligand-receptor binding, the micelles were conjugated with RGD peptide, which is known to specifically recognize integrins on cell surface [43]. Fluorescent microscopy imaging results indicated that RGD peptide-conjugated PS-PEG micellar QDs could bind with α_*v*_β_3_-integrin over-expressed cells (U87MG cell line, a human glioblastoma cell line, Fig. 6c, right), judging by the significant QD fluorescence on or in the cells. In contrast, the two control experiments, one of which used RGD peptide-conjugated PS-PEG micellar QDs to incubate with MCF cells (without α_*v*_β_3_-integrin over-expression, Fig. 6c, left) and the other of which used PS-PEG-COOH micellar QDs (without RGD peptide conjugation) to incubate with U87MG cells (Fig. 6c, middle), showed little to no QD fluorescence on or in the cells. Thus, these results demonstrated the ability of RGD peptide-conjugated PS-PEG micellar QDs to specifically bind with α_*v*_β_3_-integrin molecules.

## Conclusions

In conclusion, we have used QDs as the model nanocrystals to follow the interfacial instability process, an emerging general method to fabricate nanocrystal-encapsulated micelles. Our results reveal the key roles of emulsion droplet size and the surfactant PVA in the interfacial instability process. These results not only help to optimize the quality of nanocrystal-encapsulated micelles for biological applications such as biological detection, imaging and therapy, but offer helpful new knowledge on the interfacial instability process in particular and self-assembly in general.

## Additional Files

Additional file 1: Figure S1. Change of emulsion droplet size with increased time of mechanical treatment by (a) manual shaking, and (b) sonication, respectively. (TIF 260 kb)

Additional file 2: Figure S2. Size and morphology study of PS-PEG micellar QDs just formed by the interfacial instability method. (a) and (c) are TEM images of PS-PEG micellar QDs just formed by manual shaking for 1 min and sonication for 30 sec, respectively, in the emulsification step of the interfacial instability method. (TIF 1853 kb)

Additional file 3: Figure S3. TEM images of the top portion of PS-PEG micellar QDs samples formed by (a) manual shaking (1 min) and (b) sonication (30 sec), respectively, in the interfacial instability method after 10-day storage. (TIF 1628 kb)

Additional file 4: Figure S4. Change of fluorescent intensity of hydrophobic QDs (0.01 μM) dissolved in chloroform with increased bath sonication time. (TIF 513 kb)

Additional file 5: Figure S5. Spatial distributions of Tat peptide-conjugated PS-PEG micellar QDs (10 nM QDs) at various time points of delivery into live HeLa cells. (TIF 1129 kb)

Additional file 6: Video 1. Three dimensional reconstructured confocal images of Tat peptide-conjugated PS-PEG micellar QDs (10 nM QDs) in live HeLa cells after 24 hrs of incubation. (AVI 4217 kb)

## Abbreviations

EDC: 1-Ethyl-3-(-3-dimethylaminopropyl) carbodiimide hydrochloride
PS-PEG: Poly (styrene-b-ethylene glycol)
PS-PEG-COOH: Carboxylic acid terminated poly (styrene-b-ethylene glycol)
PTA: Phosphotungstic acid
PVA: Poly (vinyl alcohol)
QD: Quantum dot
SPION: Superparamagnetic iron oxide nanoparticle
TEM: Transmission electron microscopy
THF: Tetrahydrofuran
