# Supplementary figures and images for: Examining the Roles of Emulsion Droplet Size and Surfactant in the Interfacial Instability-Based Fabrication Process of Micellar Nanocrystals
Source: Nanoscale Res Lett. 2017 Jun 30;12:434. doi: 10.1186/s11671-017-2202-x (PMC5509569; doi:10.1186/s11671-017-2202-x)

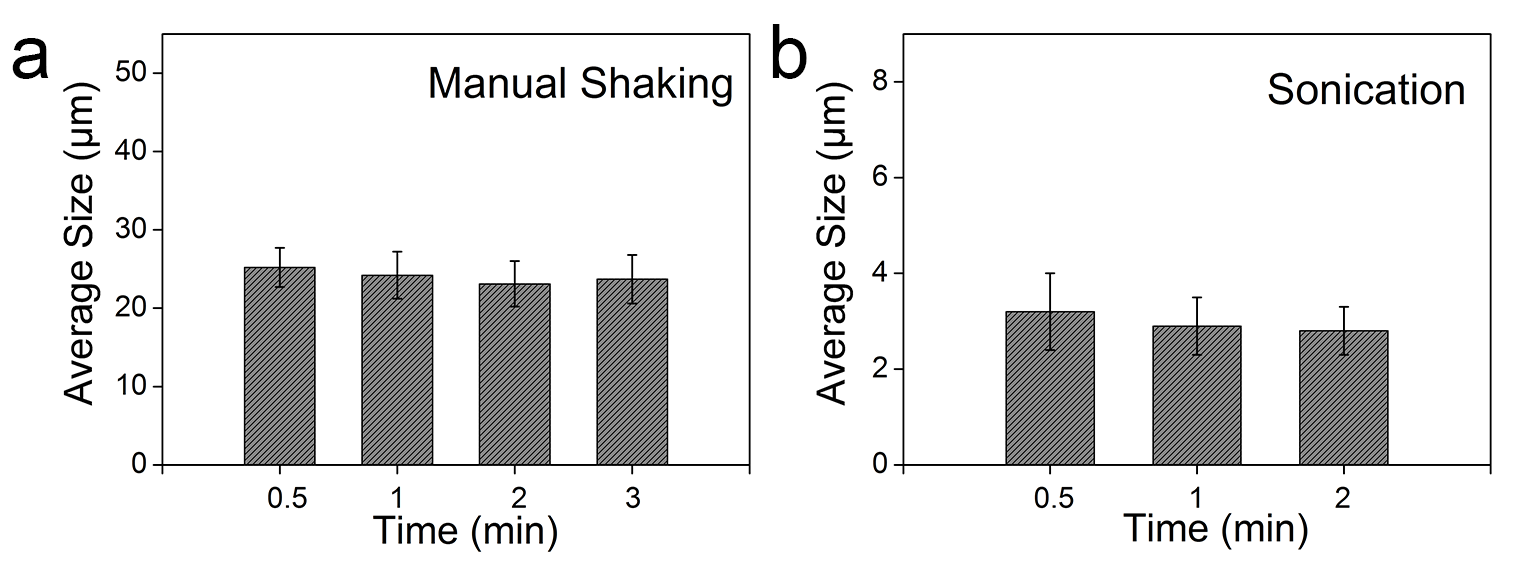

Supplement: Supplementary file 1 — Change of emulsion droplet size with increased time of mechanical treatment by (a) manual shaking, and (b) sonication, respectively. (TIF 260 kb) [file 11671_2017_2202_MOESM1_ESM.tif]

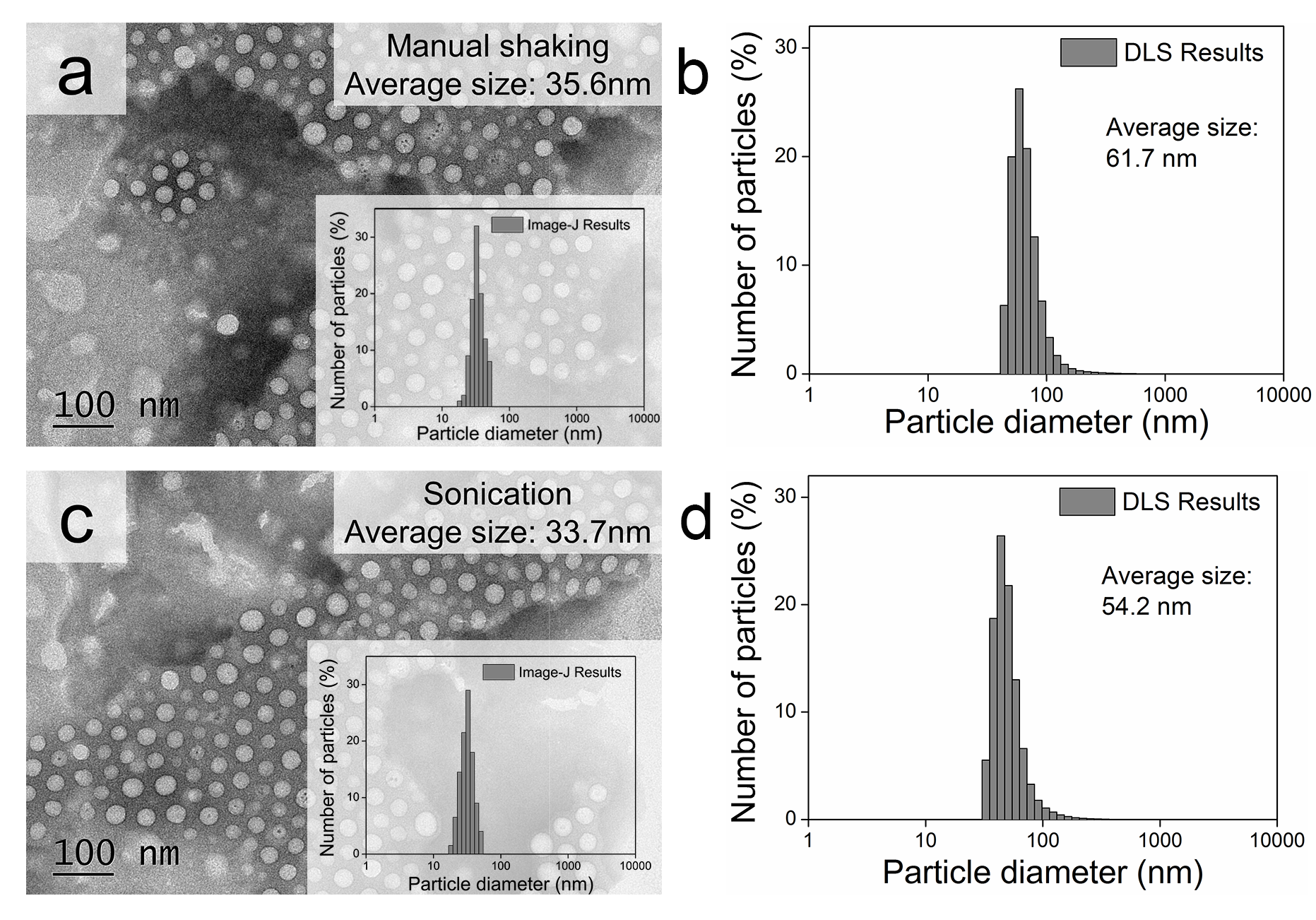

Supplement: Supplementary file 2 — Size and morphology study of PS-PEG micellar QDs just formed by the interfacial instability method. (a) and (c) are TEM images of PS-PEG micellar QDs just formed by manual shaking for 1 min and sonication for 30 sec, respectively, in the emulsification step of the interfacial instability method. (TIF 1853 kb) [file 11671_2017_2202_MOESM2_ESM.tif]

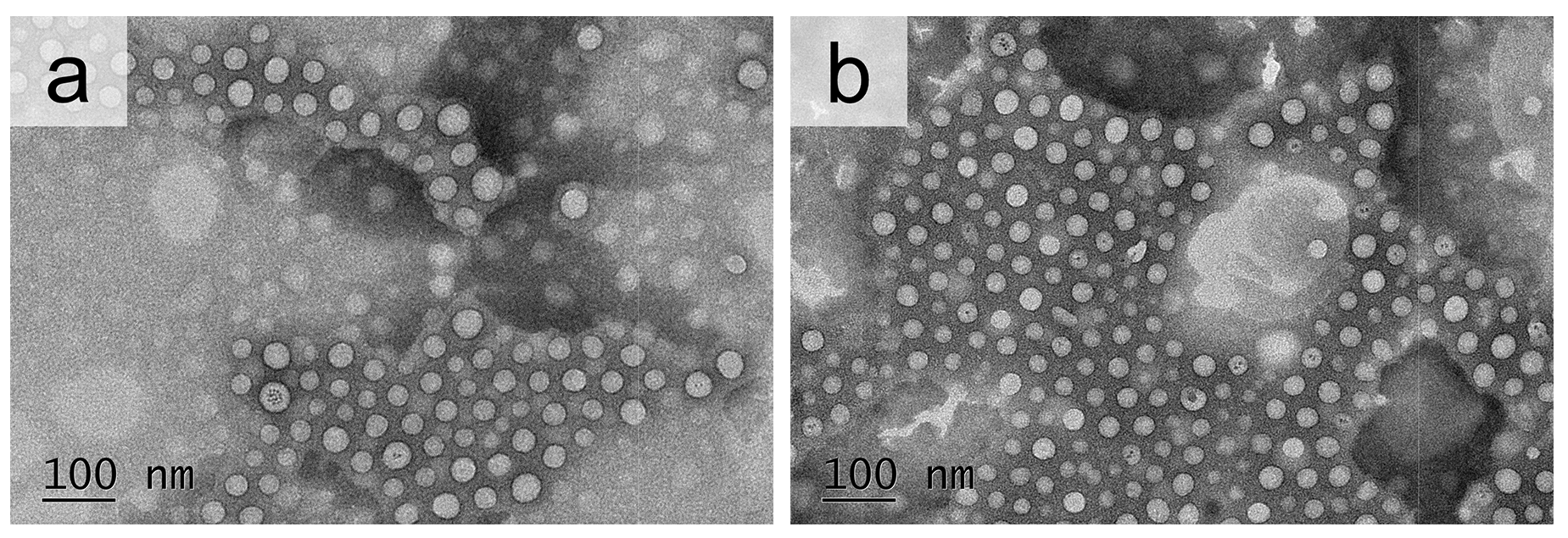

Supplement: Supplementary file 3 — TEM images of the top portion of PS-PEG micellar QDs samples formed by (a) manual shaking (1 min) and (b) sonication (30 sec), respectively, in the interfacial instability method after 10-day storage. (TIF 1628 kb) [file 11671_2017_2202_MOESM3_ESM.tif]

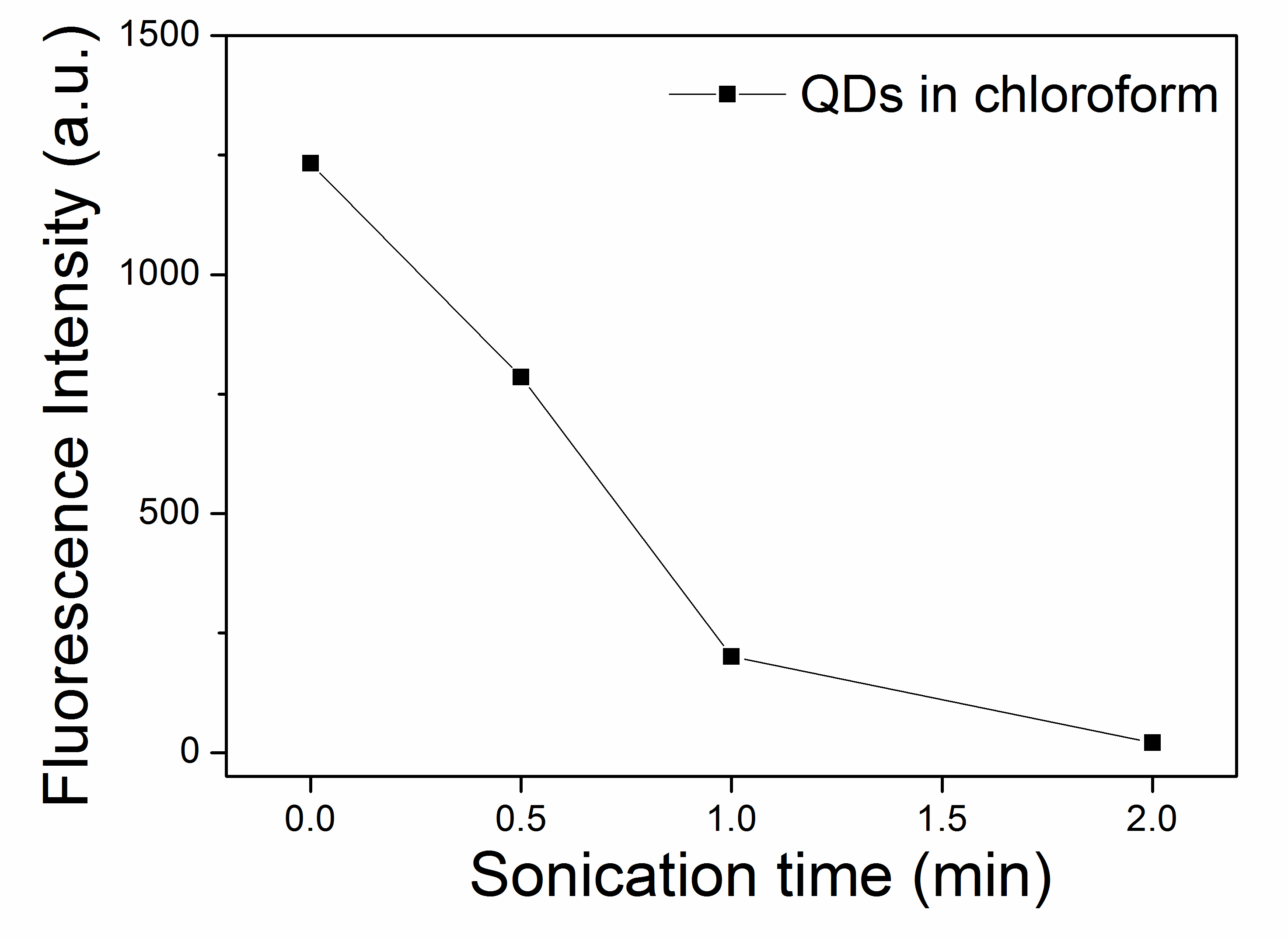

Supplement: Supplementary file 4 — Change of fluorescent intensity of hydrophobic QDs (0.01 μM) dissolved in chloroform with increased bath sonication time. (TIF 513 kb) [file 11671_2017_2202_MOESM4_ESM.tif]

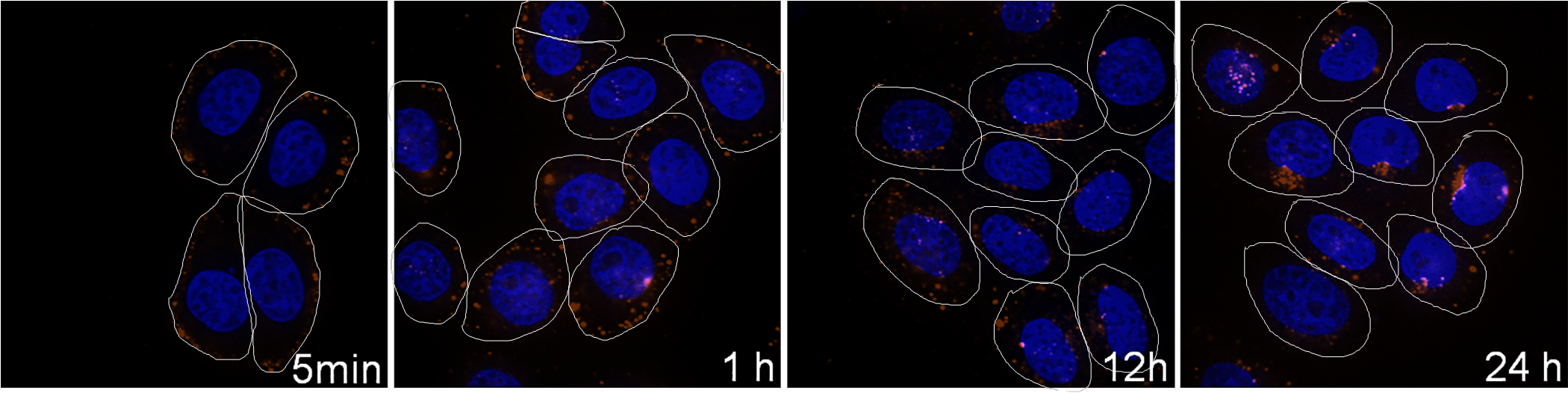

Supplement: Supplementary file 5 — Spatial distributions of Tat peptide-conjugated PS-PEG micellar QDs (10 nM QDs) at various time points of delivery into live HeLa cells. (TIF 1129 kb) [file 11671_2017_2202_MOESM5_ESM.tif]
